# Emerging regulated cell death (cuproptosis, disulfidptosis, and PANoptosis) in ischemic stroke: research progress and translational prospects

**DOI:** 10.3389/fcell.2026.1893238

**Published:** 2026-08-20

**Authors:** Dongyang Xing, Ke Xiang

**Affiliations:** 1 Department of Geriatric Medicine, Changchun University of Chinese Medicine, Jingyue National High-Tech Industrial Development Zone, Changchun, Jilin, China; 2 Department of Geriatric Medicine, The First Clinical Hospital of Jilin Provincial Academy of Chinese Medical Sciences, Changchun, Jilin, China

**Keywords:** cuproptosis, disulfidptosis, ischemic stroke, neuroinflammation, neurovascular unit, panoptosis, translational medicine

## Abstract

Ischemic stroke (IS) is one of the leading causes of disability and mortality worldwide. Its pathological mechanisms involve complex cascades including energy metabolism failure, excitotoxicity, oxidative stress, neuroinflammation, and multiple forms of regulated cell death (RCD). In recent years, cuproptosis, disulfidptosis, and PANoptosis, as three emerging RCD modalities, have attracted increasing attention in ischemic brain injury. Cuproptosis is triggered by copper overload and leads to proteotoxic stress via abnormal oligomerization of lipoylated mitochondrial proteins. Disulfidptosis occurs under glucose deprivation combined with high SLC7A11 expression, driven by NADPH depletion and intracellular disulfide stress that collapses the actin cytoskeleton. PANoptosis integrates pyroptosis, apoptosis, and necroptosis through the PANoptosome—a multiprotein platform assembled by innate immune sensors (ZBP1, AIM2, NLRP3) together with adaptor proteins (ASC, FADD) and effectors (caspase-1/8, RIPK3, MLKL, GSDMD)—leading to simultaneous execution of all three death programs and amplified neuroinflammatory injury. These three death modalities exhibit marked cell-type heterogeneity across neurons, microglia, astrocytes, and endothelial cells within the neurovascular unit, and converge on shared hubs of oxidative stress, mitochondrial dysfunction, and inflammatory signaling, forming a complex inter-pathway crosstalk network. This review systematically summarizes the molecular mechanisms, cellular specificity, spatiotemporal dynamics, and inter-pathway crosstalk of these three emerging RCDs in ischemic stroke, and discusses the clinical translational prospects of targeted and combination intervention strategies—including a stage-classified analysis of therapeutic candidates from preclinical to clinical development—providing a theoretical basis for the design of novel neuroprotective agents.

## 1 Introduction

Ischemic stroke accounts for approximately 70%–80% of all stroke cases. Its core pathological process is the interruption of cerebral blood flow leading to oxygen-glucose deprivation (OGD), which triggers cascading damage to multiple components of the neurovascular unit (NVU) (Mendelson and Prabhakaran, 2021; Hollist et al., 2021). Traditionally, necrosis was considered predominant in the ischemic core, whereas apoptosis was thought to dominate in the penumbra. However, recent studies have revealed that pyroptosis, necroptosis, ferroptosis, and autophagic cell death collectively participate in the spatiotemporal evolution of ischemic brain injury (Tuo et al., 2022; Gong et al., 2024).

**FIGURE 1:**
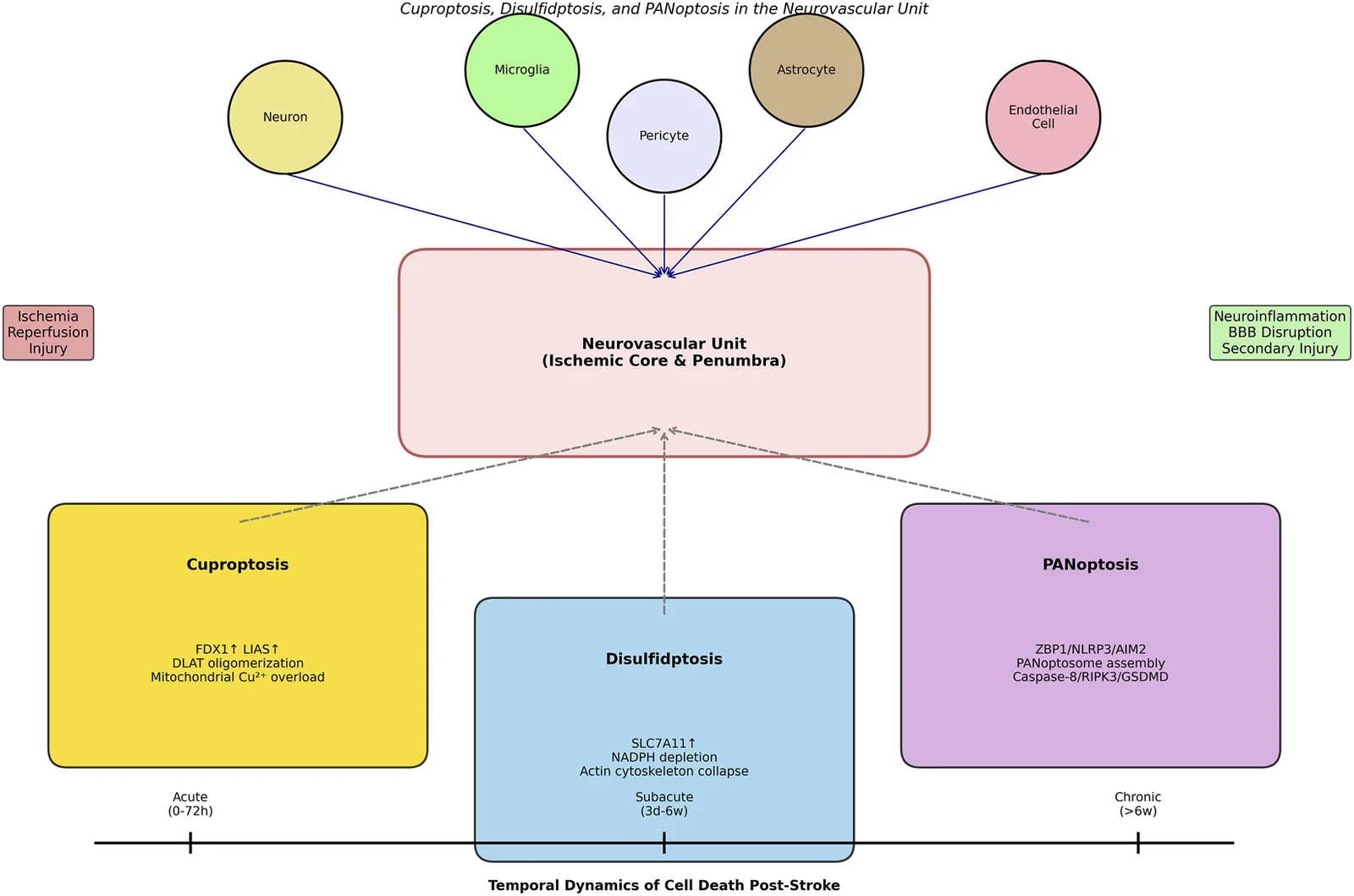
Overview of emerging regulated cell death in ischemic stroke. Ischemia-reperfusion injury triggers the spatiotemporal-specific activation of cuproptosis, disulfidptosis, and PANoptosis in the neurovascular unit (neurons, microglia, astrocytes, endothelial cells, pericytes). Based on currently available, largely indirect evidence, we propose a tentative temporal staging in which cuproptosis mainly occurs in the acute phase (0–72 h), disulfidptosis intensifies in the subacute phase (3 days–6 weeks), and PANoptosis spans from the acute to chronic phases; this temporal framework is a hypothesis-generating proposal awaiting direct longitudinal validation (see Section 5.2). The three modalities interact with each other, collectively promoting neuroinflammation, blood-brain barrier disruption, and secondary injury.

In 2022, Tsvetkov et al. (2022) first reported cuproptosis in Science—a novel form of RCD directly induced by copper ions and dependent on mitochondrial respiration. In 2023, Liu et al. (2023) revealed disulfidptosis in Nature Cell Biology—a RCD driven by disulfide stress in SLC7A11-high cells under glucose starvation. Earlier, in 2020, Kanneganti’s team (Christgen et al., 2020; Zheng and Kanneganti, 2020a) had introduced the concept of PANoptosis, emphasizing that innate immune sensors such as ZBP1, AIM2, and NLRP3 assemble the PANoptosome to simultaneously activate pyroptosis, apoptosis, and necroptosis. Research on these three emerging RCDs in ischemic stroke is still in its infancy, but accumulating evidence indicates their deep involvement in the pathological progression of cerebral ischemia-reperfusion injury (CIRI) (Wang J. et al., 2024; Xing et al., 2024; Liu et al., 2024; Tian et al., 2025). The proposed spatiotemporal relationships among these emerging RCD modalities are summarized in Figure 1.

## 2 Cuproptosis in ischemic stroke

### 2.1 Molecular mechanisms of cuproptosis

**FIGURE 2:**
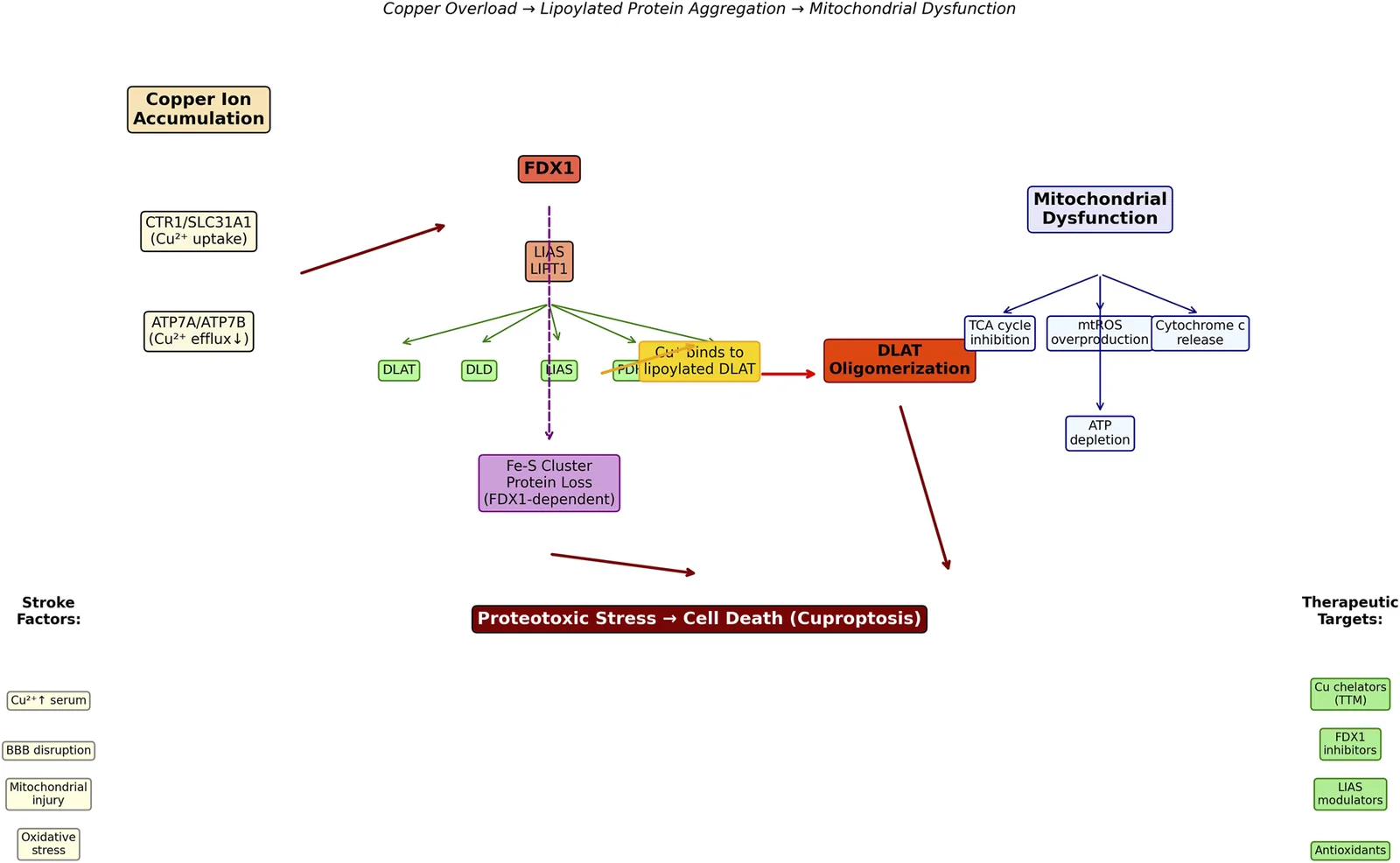
Molecular mechanisms of cuproptosis in ischemic stroke. Ischemic stroke leads to elevated serum copper and blood-brain barrier disruption, allowing copper entry via CTR1. FDX1 reduces Cu^2+^ to Cu^+^ and cooperates with LIAS/LIPT1 to mediate lipoylation of DLAT and other proteins. Cu^+^ binding to lipoylated DLAT induces its oligomerization, while Fe-S cluster protein loss occurs simultaneously. This results in TCA cycle inhibition, mitochondrial ROS overproduction, cytochrome c release, and ATP depletion, ultimately triggering proteotoxic stress and cell death (cuproptosis). Potential therapeutic targets include copper chelators (TTM), FDX1 inhibitors, LIAS modulators, and mitochondrial antioxidants.

Cuproptosis is a unique form of RCD triggered by excessive copper accumulation, distinct from apoptosis, ferroptosis, and pyroptosis (Tsvetkov et al., 2022; Chen et al., 2022). Under physiological conditions, copper enters cells via copper transporter 1 (CTR1/SLC31A1) and is exported by copper-transporting ATPases (ATP7A/ATP7B), maintaining intracellular copper homeostasis (Xie et al., 2023). During copper overload, FDX1 (ferredoxin 1) reduces Cu^2+^ to the more toxic Cu^+^, while FDX1 and LIAS (lipoic acid synthetase) mediate the lipoylation of the pyruvate dehydrogenase complex E2 subunit (DLAT) (Tsvetkov et al., 2022; Chen et al., 2023a). Cu^+^ binds to lipoylated DLAT, inducing its aberrant oligomerization and the formation of insoluble aggregates that generate proteotoxic stress. Additionally, FDX1 inhibits Fe-S cluster protein synthesis, further disrupting mitochondrial function (Tsvetkov et al., 2022; Ban et al., 2024). The molecular mechanism of cuproptosis in ischemic stroke is summarized in Figure 2.

### 2.2 Copper metabolism disturbance in ischemic stroke

Clinical studies have demonstrated that serum copper levels are significantly elevated in ischemic stroke patients compared with controls, and plasma copper is positively associated with the risk of first-ever ischemic stroke (Zhang et al., 2023; Hu et al., 2022). During CIRI, blood-brain barrier (BBB) disruption allows peripheral copper ions to infiltrate the brain parenchyma, while ischemia-hypoxia downregulates metallothionein expression, weakening intracellular copper detoxification (Chen et al., 2024). Xing et al. (2024) reported that mitochondrial oxidative stress creates conditions for cuproptosis; reducing mitochondrial ROS production and clearing damaged mitochondria are important measures to alleviate neuronal oxidative stress and cuproptosis.

### 2.3 Expression characteristics of cuproptosis-related genes

Bioinformatics analyses have revealed the expression profiles of cuproptosis-related genes (CuRGs) in ischemic stroke. Fan et al. (2022) identified differential expression of FDX1, LIAS, LIPT1, DLD, DLAT, PDHA1, PDHB, and GLS by integrating bulk and single-cell RNA-seq data. Zhang et al. (2024) further identified potential cuproptosis biomarkers in cerebral ischemia and demonstrated that CuRG expression patterns correlate with infarct severity and clinical outcome, supporting their utility as diagnostic and prognostic indicators. Jinshi et al. (2024) found that CuRGs participate in immunodeficiency following ischemic stroke, suggesting that cuproptosis may indirectly influence brain injury repair by modulating the immune microenvironment.

### 2.4 Interplay between cuproptosis and neuroinflammation

Copper homeostasis imbalance is closely linked to neuroinflammation. LIAS, as an Fe-S cluster protein, is associated with oxidative stress and inflammatory responses (Rui et al., 2023). LIAS overexpression reduces pro-inflammatory cytokines, inhibits NF-κB activity, enhances NRF2-mediated antioxidant defense, and protects mitochondrial function (Xing et al., 2024). Moreover, copper chelators such as clioquinol significantly reduce immune cell infiltration, including CD4^+^ and CD8^+^ T cells, alleviating encephalitis development (Xing et al., 2024; Chen et al., 2023a).

## 3 Disulfidptosis in ischemic stroke

### 3.1 Molecular mechanisms of disulfidptosis

Disulfidptosis was first reported by Gan and Chen’s team (Liu et al., 2023) in 2023. Its occurrence requires two key conditions: high SLC7A11 expression and glucose deprivation. SLC7A11 is the catalytic subunit of System Xc^−^, which mediates cystine influx and glutamate efflux in a 1:1 ratio (Koppula et al., 2021). Under glucose-sufficient conditions, high SLC7A11 expression promotes cystine uptake, which is reduced to cysteine via the NADPH-dependent thioredoxin system, thereby synthesizing glutathione (GSH) for antioxidant protection (Kagan et al., 2017).

**FIGURE 3:**
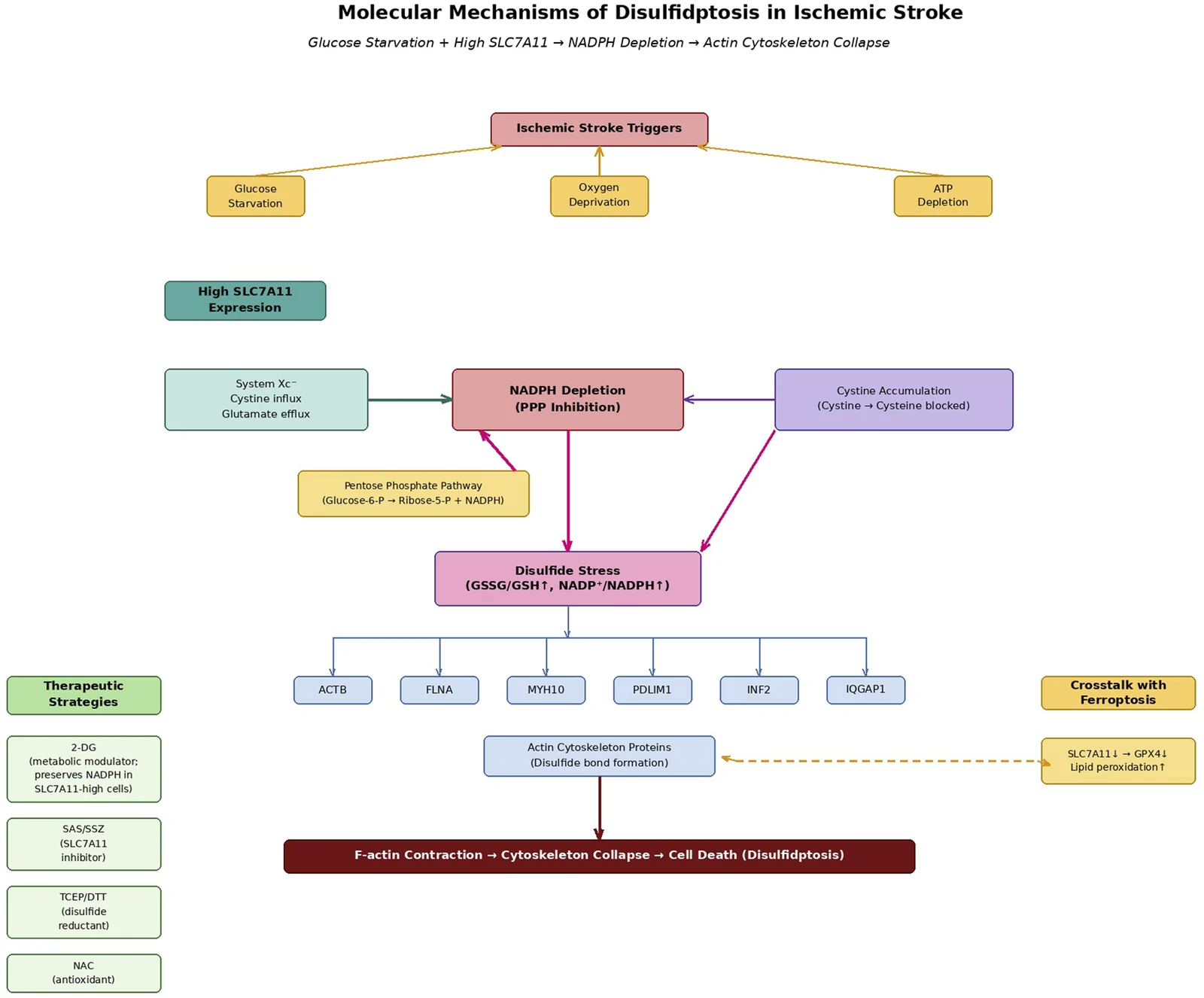
Molecular mechanisms of disulfidptosis in ischemic stroke. Ischemic stroke triggers glucose starvation, oxygen deprivation, and ATP depletion. High SLC7A11 expression promotes System Xc^−^-mediated cystine influx, but glucose deprivation suppresses the PPP pathway, leading to NADPH depletion. Cystine cannot be reduced to cysteine and accumulates intracellularly, triggering disulfide stress (elevated GSSG/GSH and NADP^+^/NADPH ratios). Aberrant disulfide bonds bind to actin cytoskeleton proteins (ACTB, FLNA, MYH10, *etc.*), causing F-actin contraction, cytoskeletal collapse, and disulfidptosis. This process interacts with ferroptosis via the SLC7A11/GPX4 axis. Therapeutic strategies include 2-DG (NADPH supply), SAS (SLC7A11 inhibitor), TCEP/DTT (disulfide reductants), and NAC (antioxidant).

However, under glucose deprivation, the pentose phosphate pathway (PPP) is suppressed and NADPH generation is sharply reduced (Zhang and Peng, 2014). High SLC7A11 expression then leads to massive intracellular cystine accumulation. Without sufficient NADPH to reduce cystine to cysteine, disulfide stress ensues (Liu et al., 2023). Accumulated cystine forms aberrant disulfide bonds, particularly with actin cytoskeleton proteins (ACTB, FLNA, MYH10, PDLIM1, INF2, IQGAP1), causing F-actin contraction, cytoskeletal collapse, and cell death (Liu et al., 2023; Xiao et al., 2024). Notably, disulfidptosis cannot be rescued by conventional apoptosis, necroptosis, ferroptosis, or autophagy inhibitors, but can be prevented by disulfide reductants (TCEP, DTT, β-mercaptoethanol) or SLC7A11 inhibitors (sulfasalazine, SAS) (Liu et al., 2023). The molecular mechanism of disulfidptosis in ischemic stroke is summarized in Figure 3.

### 3.2 Triggering factors in ischemic stroke

Ischemic stroke provides an ideal pathological environment for disulfidptosis. Vascular occlusion causes simultaneous glucose and oxygen deprivation, impairing mitochondrial oxidative phosphorylation and exacerbating bioenergetic stress (An et al., 2021; Sharma et al., 2022). Clinical studies show that serum homocysteine and cysteine concentrations are significantly elevated during the acute phase of stroke (Salemi et al., 2009). Animal experiments confirm that cysteine levels in the hippocampus and striatum increase 10- to 13-fold within 8 h after ischemia, mainly derived from GSH breakdown (Slivka and Cohen, 1993). Qu et al. (Qu et al., 2006) found that GSH-derived cysteine accumulation significantly increases excitotoxic injury and disrupts the GSH/GSSG balance, exacerbating tissue damage. It should be noted that the direct trigger of disulfidptosis is intracellular cystine (the oxidized disulfide form) rather than cysteine itself; the above clinical and animal observations of elevated cysteine and GSH catabolism therefore provide indirect evidence by indicating disrupted thiol/disulfide homeostasis and a likely shift in the GSSG/GSH and cystine/cysteine ratios under post-ischemic NADPH depletion. Direct *in vivo* quantification of intracellular cystine and NADP^+^/NADPH ratios in the post-ischemic brain remains an outstanding need.

### 3.3 Disulfidptosis-related genes and diagnostic value

Zhao et al. (2024) systematically identified disulfidptosis-related genes (DRGs) in ischemic stroke by combining single-cell sequencing, machine learning algorithms, and *in vitro* experiments. Integrating the GSE16561 and GSE58294 datasets, they found that SLC7A11 was significantly upregulated in the ischemic stroke group, whereas NDUFA11 was downregulated (Zhao et al., 2024). Using LASSO regression, SVM-RFE, and random forest algorithms, five key DRGs (SLC7A11, RPN1, NUBPL, NDUFA11, and NDUFS1) were selected to construct a predictive model with high diagnostic accuracy (Zhao et al., 2024). Single-cell analysis showed that these DRGs are mainly distributed in immune cell types, suggesting that disulfidptosis may specifically regulate neuroimmune interactions (Qin et al., 2023).

### 3.4 Crosstalk between disulfidptosis and ferroptosis

SLC7A11 is the critical node connecting disulfidptosis and ferroptosis. In ferroptosis, SLC7A11-mediated cystine uptake is the rate-limiting step for GSH synthesis, and GSH is an essential cofactor for GPX4 (glutathione peroxidase 4) (Dixon et al., 2012). GPX4 utilizes GSH to reduce toxic lipid peroxides to non-toxic lipid alcohols, thereby inhibiting ferroptosis (Ingold et al., 2018). In ischemic stroke, massive glutamate release reverses the physiological transport gradient of System Xc^−^, severely impairing cystine uptake and causing intracellular cysteine depletion and GPX4 inactivation, ultimately triggering ferroptosis (Bridges et al., 2012).

However, when SLC7A11 is highly expressed under glucose deprivation, the same transporter paradoxically drives disulfidptosis (Liu et al., 2023; Zhao et al., 2024). This ‘double-edged sword’ effect suggests that SLC7A11 regulation must consider the metabolic context: under energy sufficiency, high SLC7A11 expression is protective; under glucose starvation, it becomes lethal (Zhao et al., 2024). Critically, existing studies diverge on the threshold glucose concentrations at which this switch occurs: *in vitro* data from Liu et al. (2023) indicate that disulfidptosis is triggered below approximately 1 mM glucose in SLC7A11-overexpressing cells, whereas *in vivo* ischemic penumbra glucose levels range from 0.2 to 2.5 mM depending on collateral perfusion—a gradient that may explain why disulfidptosis is spatially restricted rather than uniform across the ischemic territory. We therefore interpret the available evidence as supporting a metabolic-state-first model: the net functional outcome of SLC7A11 activity is determined primarily by cellular NADPH/NADP^+^ ratio and glucose flux, rather than by expression level alone. This interpretive position has direct therapeutic implications, as indiscriminate SLC7A11 inhibition during the acute phase risks exacerbating ferroptosis in the penumbra. Recent studies also found that copper ions can directly bind to cysteine residues (C107 and C148) of GPX4, promoting GPX4 ubiquitination and autophagic degradation, thereby enhancing ferroptosis sensitivity (Xue et al., 2023), further revealing the interplay between cuproptosis and ferroptosis.

### 3.5 PRDX1 and ischemic postconditioning

Peroxiredoxin 1 (PRDX1) is a key molecule in the disulfidptosis regulatory network. Liu et al. (2024) found that ischemic postconditioning (IPostC) exerts neuroprotective effects by regulating PRDX1-mediated disulfidptosis-related pathways. Bioinformatics analysis combined with Mendelian randomization confirmed that PRDX1 shares causal variant locus rs17522918 with ischemic stroke, and PRDX1-related methylation sites (cg02631906 and cg08483560) are causally associated with ischemic stroke risk (Liu et al., 2024).

## 4 PANoptosis in ischemic stroke

### 4.1 Concept and molecular architecture of PANoptosis

**FIGURE 4:**
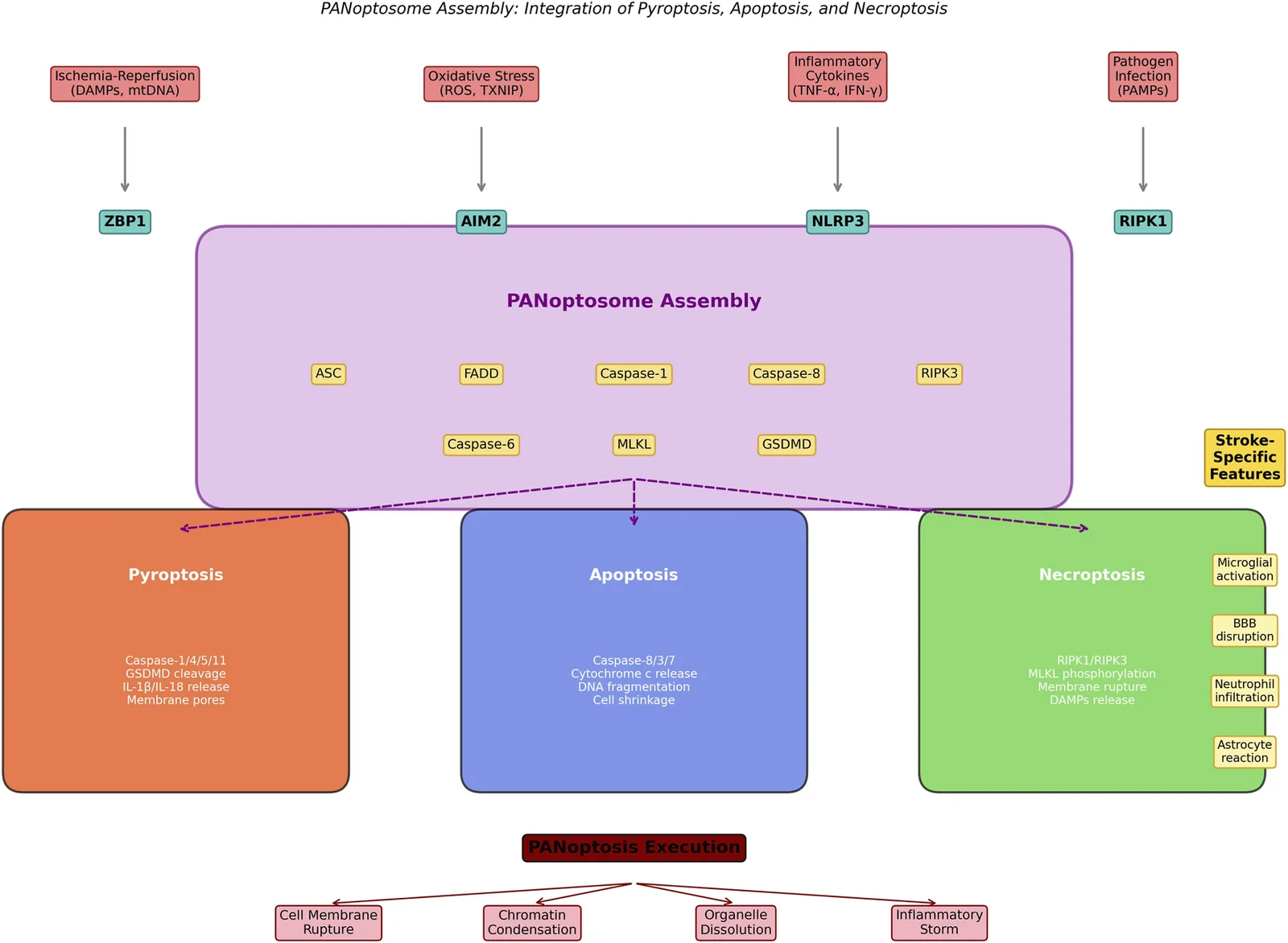
Molecular mechanisms of PANoptosis in ischemic stroke. Ischemia-reperfusion injury releases DAMPs (e.g., mtDNA), ROS/TXNIP, and cytokines (TNF-α/IFN-γ), activating sensors ZBP1, AIM2, NLRP3, and RIPK1. These assemble the PANoptosome (ASC, FADD, caspase-1/8, RIPK3, caspase-6, MLKL, GSDMD) to synchronously execute: (1) Pyroptosis via GSDMD cleavage and IL-1β/IL-18 release; (2) Apoptosis via caspase-8/3/7 activation; (3) Necroptosis via MLKL phosphorylation and membrane rupture. Stroke-specific features include microglial activation, BBB disruption, and inflammatory amplification. Solid arrows indicate experimentally supported interactions; dashed arrows represent hypothetical connections pending validation.

PANoptosis was proposed by Kanneganti’s team (Christgen et al., 2020; Zheng and Kanneganti, 2020a) in 2020 and is defined as synergistic cell death simultaneously activating pyroptosis, apoptosis, and necroptosis. Its unique feature is the PANoptosome—a multimeric protein complex that integrates molecular components of the three death pathways for synchronous regulation (Place et al., 2021). The core structure of the PANoptosome includes sensors (ZBP1, AIM2, NLRP3, RIPK1, NLRP12, NLRC5, RIG-I), adaptor proteins (ASC, FADD), and effector molecules (caspase-1/8, RIPK3, MLKL, GSDMD) (Wu et al., 2025; Sharma et al., 2023). The core PANoptosome architecture and the integrated execution of pyroptosis, apoptosis, and necroptosis are illustrated in Figure 4.

### 4.2 ZBP1-PANoptosome in cerebral ischemia

ZBP1 (Z-DNA binding protein 1, also known as DAI) is a key upstream sensor of PANoptosis. Its N-terminus contains two Zα domains that recognize Z-DNA/Z-RNA, and its RHIM domain interacts with RHIM-containing proteins such as RIPK1 and RIPK3 (Kuriakose and Kanneganti, 2018). During CIRI, mitochondrial DNA (mtDNA) leaks into the cytoplasm, forming Z-form nucleic acids that activate ZBP1 (Wan et al., 2026). ZBP1 recruits RIPK3 via the RHIM domain to promote MLKL phosphorylation and drive necroptosis; simultaneously, it activates apoptosis through the FADD-caspase-8 axis and pyroptosis through the ASC-NLRP3-caspase-1 axis (Zheng and Kanneganti, 2020b). Caspase-6, as an important component of the ZBP1-PANoptosome, enhances the interaction between RIPK3 and ZBP1 through a non-enzymatic function, promoting efficient PANoptosome assembly (Zheng et al., 2020).

Wan et al. (2026) found in a retinal ischemia-reperfusion injury model that VDAC1 oligomerization regulates PANoptosis, suggesting that the mitochondrial permeability transition pore (mPTP) may serve as a bridge connecting energy crisis to PANoptosis activation. In brain ischemia, OGD leads to ATP depletion, Na^+^/K^+^-ATPase failure, neuronal depolarization, and release of glutamate and ROS, further triggering death ligand-receptor interactions that activate FADD-mediated apoptosis or TRADD-mediated necroptosis (Tian et al., 2025).

### 4.3 AIM2-PANoptosome and NLRP3-PANoptosome

AIM2 (absent in melanoma 2) is a cytosolic sensor that recognizes double-stranded DNA and forms the AIM2-PANoptosome during Francisella novicida and HSV-1 infection (Man et al., 2020). AIM2 cooperates with ZBP1 to recruit ASC and caspase-1 to form the inflammasome, driving GSDMD cleavage and pyroptosis; simultaneously, it activates caspase-3/7-mediated apoptosis and induces MLKL-dependent necroptosis (Lee et al., 2021). In ischemic stroke, extracellular DNA released from damaged neurons and glial cells can be recognized by AIM2, triggering PANoptosis (Zhang et al., 2025a).

The NLRP3 inflammasome is the most extensively studied inflammatory complex in brain ischemia. After ischemia-reperfusion, mitochondrial ROS production, K^+^ efflux, lysosomal rupture, and TXNIP de-repression collectively activate NLRP3 (Qi et al., 2026). NLRP3 recruits ASC and caspase-1, cleaves GSDMD to release its N-terminal domain, forms pores on the membrane, and drives pyroptosis and IL-1β/IL-18 maturation (Swanson et al., 2019). Notably, NLRP3 is not only a platform for pyroptosis but can also interact with apoptosis and necroptosis pathways through the PANoptosome (Malireddi et al., 2020). Tian et al. (2025) systematically reviewed the interplay between PANoptosis and autophagy in ischemic stroke, noting that autophagy can negatively regulate PANoptosis by clearing damaged mitochondria and inflammasome components, but excessive autophagy may also promote death through Beclin-1/caspase-8 interactions.

### 4.4 Cell specificity of PANoptosis in ischemic stroke

Yan et al. (2022) first confirmed the existence of PANoptosis in CIRI through bibliometrics, data mining, and multi-dimensional analysis. In OGD/R cell models and tMCAO animal models, TUNEL staining (DNA fragmentation, indicative of apoptosis), EthD-III, and PI staining (both reporting loss of plasma-membrane integrity characteristic of necroptotic and pyroptotic cell death) together demonstrated the simultaneous occurrence of features attributable to the three death programs, which was further dissected with pathway-specific inhibitors below (Yan et al., 2022). Pathway-specific inhibitors (Z-VAD-FMK, disulfiram, Nec-1s) alone only partially reduced cell death, whereas triple inhibitors significantly decreased the TUNEL-positive rate (from 40% to 15%), confirming the synergistic lethal effect of PANoptosis (Yan et al., 2022).

At the cellular level, PANoptosis exhibits significant heterogeneity: (1) Neurons: After OGD, neurons mainly undergo apoptosis and necroptosis, but when accompanied by NLRP3 inflammasome activation and GSDMD cleavage, PANoptosis is constituted (Radak et al., 2017). Naito et al. (2020) confirmed that necroptosis and apoptosis are sequentially activated after stroke and synergistically mediate vascular and neural pathology. (2) Microglia: Ischemia rapidly activates microglia. M1 microglia highly express NLRP3, caspase-1, and GSDMD, driving pyroptosis and releasing IL-1β/IL-18, which disrupts the BBB (Dong et al., 2023). Chen et al. (2019) found that microglia-derived TNF-α mediates endothelial cell necroptosis through the TNFR1/RIPK1 pathway, exacerbating BBB disruption. (3) Astrocytes: Reactive astrocytes release ATP and glutamate through Cx43 hemichannels, activating microglial P2X7 receptors and maintaining inflammasome activity (Liao et al., 2024). Zhuang et al. (2025) reported that CD73 overexpression attenuates GSDMD-mediated astrocyte pyroptosis through the A2b/NF-κB pathway. (4) Endothelial cells: Brain microvascular endothelial cells (BMECs) undergo pyroptosis and necroptosis under oxidative stress and inflammatory attack after ischemia, leading to increased BBB permeability (Wang S. et al., 2024).

### 4.5 Interplay between PANoptosis and autophagy

Autophagy and PANoptosis exhibit complex bidirectional regulation. On one hand, autophagy inhibits ZBP1/NLRP3 activation and PANoptosome assembly by clearing damaged mitochondria and reducing mtDNA leakage and ROS production via mitophagy (Tian et al., 2025). On the other hand, autophagy-related proteins (e.g., Beclin-1, ATG5, ATG7) can participate in death regulation through non-autophagic functions. Beclin-1 dissociation from Bcl-2 promotes autophagy, but caspase-3-cleaved Beclin-1 fragments can translocate to mitochondria to trigger apoptosis (Tian et al., 2025). In ischemic stroke, the net effect of this interplay depends on the injury phase and cell type: autophagy may be protective in the acute phase but may promote PANoptosis in the chronic phase (Mo et al., 2020).

## 5 Crosstalk among emerging RCDs

### 5.1 Interplay between cuproptosis and ferroptosis

Cuproptosis and ferroptosis interact deeply at the levels of oxidative stress and mitochondrial metabolism. Both depend on mitochondrial activity: cuproptosis requires an active TCA cycle to generate lipoylated substrates, while ferroptosis depends on mitochondrial ROS-driven lipid peroxidation (Li et al., 2025). GSH is a common convergence point: GSH can chelate free copper ions to reduce DLAT lipoylation-induced protein aggregation, and is also an essential cofactor for GPX4 to inhibit ferroptosis (Liu and Chen, 2024). Studies have found that the copper ionophore elesclomol-induced copper accumulation can generate massive ROS through Fenton reactions while promoting SLC7A11 degradation and GPX4 ubiquitination, enhancing ferroptosis sensitivity (Xue et al., 2023; Gao et al., 2021). FDX1, as the core regulator of cuproptosis, is positively correlated with autophagy marker gene expression, suggesting that FDX1-mediated cuproptosis may interact with ferroptosis through autophagy (Li et al., 2025).

### 5.2 Metabolic switch between disulfidptosis and ferroptosis

SLC7A11 is the key molecular switch between disulfidptosis and ferroptosis. Under energy sufficiency, high SLC7A11 expression promotes cystine uptake and GSH synthesis, maintains GPX4 activity, and inhibits ferroptosis (Dixon et al., 2012). However, under glucose deprivation, the same high SLC7A11 expression leads to NADPH depletion and cystine accumulation, driving disulfidptosis (Liu et al., 2023). It is important to acknowledge that the proposed temporal staging model—in which disulfidptosis predominates in the subacute phase (3 days–6 weeks)—is currently a hypothesis-generating framework rather than an empirically established timeline. The cited evidence provides only indirect temporal support, and direct longitudinal measurements of disulfidptosis biomarkers (NADP^+^/NADPH ratio, actin cytoskeletal integrity, SLC7A11 spatial expression) across defined post-ischemic time points *in vivo* are lacking. Future studies employing spatially resolved metabolomics and live-cell imaging will be required to validate or refine these temporal boundaries. This metabolic-dependent switching suggests that in different regions of ischemic stroke (severe glucose deprivation in the core vs. relative preservation in the penumbra) and different time windows (acute energy crisis vs. subacute metabolic recovery), SLC7A11 may exert diametrically opposite effects (Zhao et al., 2024). Precise spatiotemporal regulation of SLC7A11 activity, or combined targeting of GPX4 and NADPH metabolism, may be key to optimizing neuroprotective strategies.

### 5.3 Potential interplay between PANoptosis and cuproptosis/disulfidptosis

Direct evidence is currently limited, but multiple potential interaction nodes exist: (1) Oxidative stress convergence: All three death modalities are regulated by ROS. Cu^+^ generates ROS through Fenton reactions in cuproptosis; NADPH depletion weakens antioxidant capacity in disulfidptosis; and mtROS activates NLRP3 and ZBP1 in PANoptosis (Chen et al., 2018). (2) Mitochondrial dysfunction: Cuproptosis directly disrupts the TCA cycle and mitochondrial membrane potential; cytoskeletal collapse in disulfidptosis may indirectly affect mitochondrial dynamics; and mPTP opening and mtDNA release are critical for PANoptosome activation (Bonora et al., 2012). (3) Inflammatory amplification: PANoptosis releases IL-1β/IL-18 and DAMPs, which further activate microglia, promote copper uptake protein or SLC7A11 expression, and form a positive feedback loop (Long et al., 2023). (4) Caspase-8 hub: Caspase-8 is a core component of the PANoptosome and participates in apoptosis and pyroptosis regulation. Recent studies have found that caspase-8 can cleave GPX4 to promote ferroptosis (Fritsch et al., 2019), suggesting it may also serve as a bridge connecting PANoptosis to ferroptosis/cuproptosis.

## 6 Therapeutic strategies and translational prospects

**FIGURE 5:**
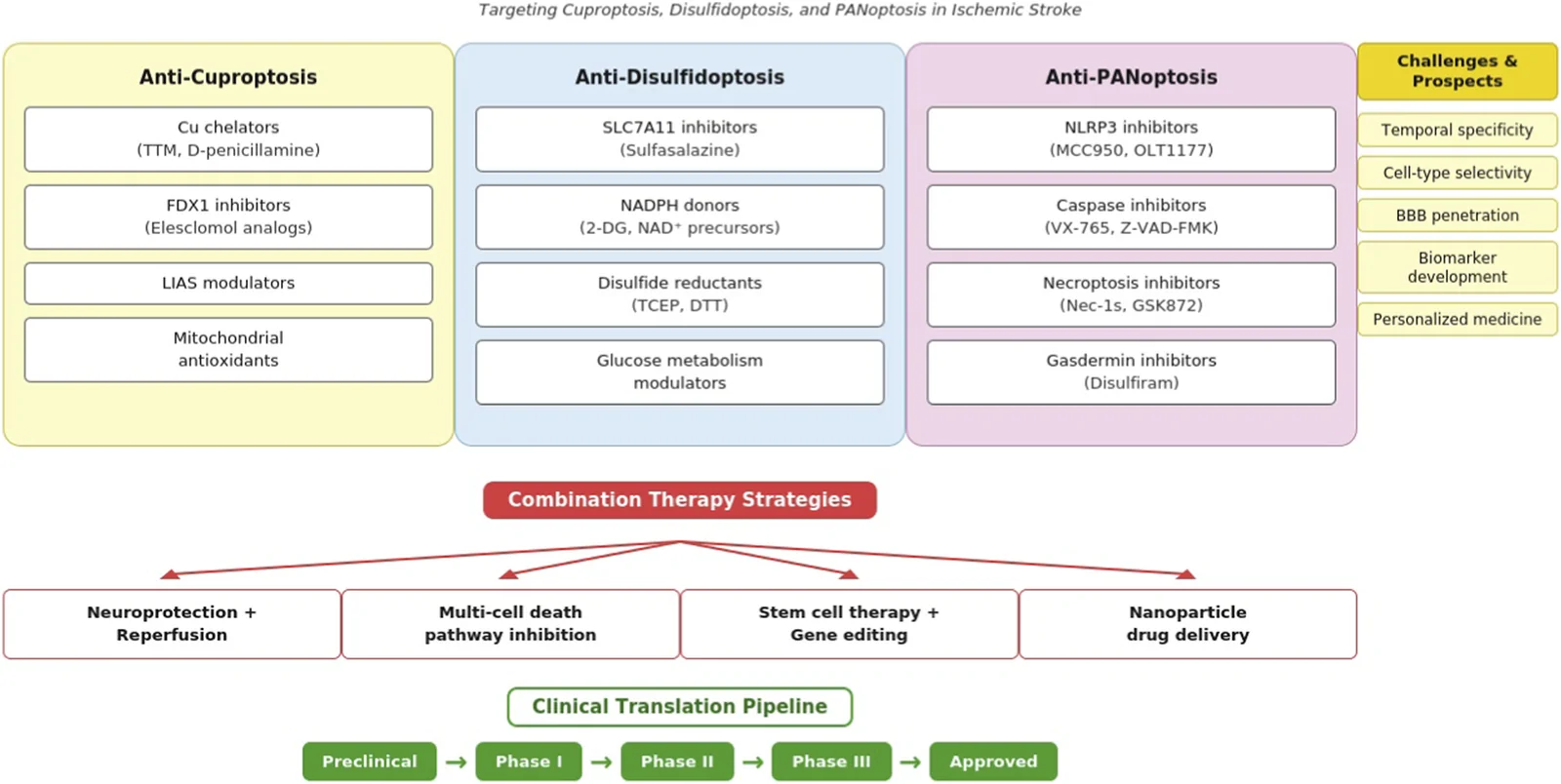
Therapeutic strategies targeting cuproptosis, disulfidptosis, and PANoptosis in ischemic stroke, classified by development stage (see Table 1). Anti-cuproptosis: copper chelators (TTM, D-penicillamine; preclinical in IS), FDX1 inhibitors, LIAS modulators. Anti-disulfidptosis: SLC7A11 inhibitors (SAS; phase-dependent use), NADPH supply agents (2-DG, NAC), disulfide reductants (TCEP, DTT). Anti-PANoptosis: NLRP3 inhibitors (MCC950 preclinical; OLT1177 Phase II for other indications), caspase inhibitors (VX-765), necroptosis inhibitors (Nec-1s), gasdermin inhibitors (disulfiram). Combination strategies encompass reperfusion plus neuroprotection, multi-RCD inhibition, stem cell therapy with gene editing, and nanoparticle-based BBB-crossing delivery.

The principal therapeutic strategies and translational pipeline are summarized in Figure 5.

### 6.1 Anti-cuproptosis strategies

Copper chelators are the most direct means of inhibiting cuproptosis. Ammonium tetrathiomolybdate (ATTM/TTM), a copper chelator used for Wilson’s disease, has been confirmed to possess hydrogen sulfide donor properties and significantly improves neurological function, reduces infarct volume, enhances antioxidant enzyme activity, and reduces oxidative damage in tMCAO rat models (Mendonca et al., 2020; Wang et al., 2018). TTM achieves ‘metabolic regulation’ by inhibiting mitochondrial cytochrome c oxidase and reducing reperfusion-driven excessive ROS production, rather than completely suppressing mitochondrial respiration (Mendonca et al., 2020). In addition, D-penicillamine and clioquinol have also shown copper chelation potential in neurodegenerative diseases (Xing et al., 2024). It must be noted, however, that neither TTM nor D-penicillamine has been evaluated in published clinical trials in ischemic stroke patients; their current evidence base is entirely preclinical (see Table 1). BBB penetration by TTM remains incompletely characterized, and the narrow therapeutic window between copper chelation and essential metal depletion poses a pharmacokinetic challenge requiring dose-optimization studies before clinical translation.

**TABLE 1:** Development stage classification of key therapeutic agents targeting emerging RCDs in ischemic stroke.

Agent	Target	RCD pathway	Current stage	Stroke-specific notes
TTM/ATTM	Copper chelation/CcO	Cuproptosis	Preclinical	No IS clinical trial data; BBB penetration uncharacterized
D-penicillamine	Copper chelation	Cuproptosis	Approved (Wilson’s disease)	No IS clinical trial data; off-label use only
Clioquinol	Copper chelation	Cuproptosis	Preclinical (neuro)	Limited CNS pharmacokinetics data
Sulfasalazine (SAS)	SLC7A11 inhibitor	Disulfidptosis/Ferroptosis	Approved (IBD/RA)	Risk of worsening ferroptosis in acute phase; phase-dependent use required
TCEP/DTT	Disulfide reductant	Disulfidptosis	Research tool only	Not suitable for systemic clinical use; alternative delivery required
NAC	GSH precursor/antioxidant	Disulfidptosis/Oxidative stress	Approved (acetaminophen toxicity)	IV formulation available; stroke-specific data limited
2-DG	metabolic modulator reported to preserve NADPH in SLC7A11-high cells (PPP)	Disulfidptosis	Phase I/II (oncology)	Hypoglycemia risk; stroke-specific dosing unstudied
MCC950	NLRP3 inhibitor	PANoptosis/Pyroptosis	Clinical development discontinued because of liver-toxicity concerns; no ischemic-stroke clinical trial.	CNS/plasma ratio <0.1; nanodelivery strategies under development
OLT1177	NLRP3 inhibitor	PANoptosis/Pyroptosis	Phase II (osteoarthritis)	Oral bioavailability confirmed; IS BBB penetration under evaluation
CY-09	NLRP3 NACHT domain	PANoptosis/Pyroptosis	Preclinical	Selective; IS-specific data lacking
VX-765 (Belnacasan)	Caspase-1 inhibitor	PANoptosis/Pyroptosis	Phase II (epilepsy)	Oral; CNS penetration demonstrated in epilepsy models
Necrostatin-1s (Nec-1s)	RIPK1 kinase inhibitor	PANoptosis/Necroptosis	Preclinical	Brain-penetrant in rodent models; no clinical data
GSK872	RIPK3 kinase inhibitor	PANoptosis/Necroptosis	Preclinical	Research tool only; no clinical data
Disulfiram	GSDMD pore inhibitor	PANoptosis/Pyroptosis	Approved (alcohol dependence)	Repurposing candidate; IS clinical data lacking; hepatotoxicity risk

FDX1 and LIAS regulation represents a more specific strategy. Elesclomol, as a copper ionophore, induces cuproptosis in cancer, but its analogs may be engineered into FDX1 inhibitors to block the lipoylation process (Calderon-Aparicio et al., 2015). LIAS overexpression enhances antioxidant defense, reduces pro-inflammatory factors, and protects mitochondrial function (Rui et al., 2023). However, brain delivery and cell specificity of these targets remain to be addressed.

### 6.2 Anti-disulfidptosis strategies

SLC7A11 inhibitors such as sulfasalazine (SAS) can block System Xc^−^ activity and significantly rescue glucose deprivation-induced cell death *in vitro* (Liu et al., 2023; Pardieu et al., 2022). However, in ischemic stroke, cautious use is required because SLC7A11 inhibition during the acute phase may simultaneously promote ferroptosis (Zhao et al., 2024). Zhao et al. (2024) found that SLC7A11 expression inhibits microglial M1 polarization and promotes M2 polarization, suggesting an anti-inflammatory effect. Therefore, SLC7A11 regulation must consider the phase: its function may need to be maintained during the acute phase to prevent ferroptosis, while moderate inhibition during the subacute phase may block disulfidptosis (Zhao et al., 2024).

NADPH supplementation strategies include 2-deoxy-D-glucose (2-DG), a glucose analog that can generate NADPH through the PPP to protect SLC7A11-high cells from disulfidptosis (Liu et al., 2023). NAD^+^ precursors such as nicotinamide riboside may also provide protection by enhancing NADPH regeneration (Imai and Guarente, 2014). Disulfide reductants such as TCEP (tris(2-carboxyethyl)phosphine) and DTT (dithiothreitol) can directly reduce aberrant disulfide bonds and rescue actin cytoskeletal integrity (Liu et al., 2023). N-acetylcysteine (NAC), as an antioxidant and GSH precursor, may also alleviate disulfidptosis by maintaining redox balance (Rushworth and Megson, 2014).

### 6.3 Anti-PANoptosis strategies

NLRP3 inflammasome inhibitors are the most extensively studied PANoptosis intervention targets. MCC950 (CRID3) is a highly selective NLRP3 inhibitor that has demonstrated neuroprotective effects in multiple brain injury models (Coll et al., 2019). OLT1177 (dapansutrile) is an orally active NLRP3 inhibitor that has completed Phase II trials for osteoarthritis, and its BBB penetration in stroke is under evaluation (Marchetti et al., 2018). CY-09 directly binds to the NACHT domain of NLRP3 to inhibit its ATPase activity (Jiang et al., 2017).

Caspase inhibitors include VX-765 (Belnacasan), an oral caspase-1 inhibitor that reduces IL-1β maturation and GSDMD cleavage (Wannamaker et al., 2007). Z-VAD-FMK is a broad-spectrum caspase inhibitor but may increase necroptosis risk due to apoptosis blockage, necessitating combination with necroptosis inhibitors (Nikoletopoulou et al., 2013). Necroptosis inhibitors include Necrostatin-1s (Nec-1s, RIPK1 kinase inhibitor) and GSK872 (RIPK3 inhibitor) (Degterev et al., 2008). Notably, necroptosis inhibition alone may shift cells toward apoptosis or pyroptosis, so combined targeting of multiple PANoptosome components may be more effective (Zhang et al., 2025b).

Gasdermin inhibitors such as disulfiram can inhibit GSDMD pore formation, reducing IL-1β release and cell lysis (Hu et al., 2020). Necrosulfonamide blocks the interaction between GSDMD and MLKL, potentially simultaneously inhibiting pyroptosis and necroptosis execution (Yang et al., 2022).

### 6.4 Combination therapy and precision medicine

The pathological complexity of ischemic stroke dictates the limitations of single-target intervention. Combination strategies include: (1) Neuroprotectants plus reperfusion: Edaravone (a free radical scavenger) combined with intravenous thrombolysis or endovascular thrombectomy improved 90-day functional outcomes in the TASTE trial (Fu et al., 2024). (2) Multi-RCD pathway inhibition: Simultaneous targeting of apoptosis (Z-VAD-FMK), pyroptosis (MCC950), and necroptosis (Nec-1s) in triple combination significantly reduced cell mortality compared with monotherapy in OGD models (Yan et al., 2022). (3) Stem cell therapy plus gene editing: Mesenchymal stem cells (MSCs) and neural stem cells (NSCs) not only provide cell replacement but also regulate the immune microenvironment through paracrine effects (Cha et al., 2024). Combined with CRISPR/dCas9 targeting neuroprotective genes such as SIRT1, endogenous repair can be enhanced (Ryu et al., 2024). hNPC01 (iPSC-derived forebrain neural progenitor cells) has received FDA and China CDE clinical trial approval (Hopstem Biotechnology, 2024). (4) Nanoparticle drug delivery: Engineered nanocarriers (e.g., PLGA nanoparticles, exosomes) can achieve BBB penetration and ischemic region-targeted delivery, improving drug bioavailability and reducing systemic side effects (Lv et al., 2024).

### 6.5 Challenges and prospects for clinical translation

Despite the rich targets provided by emerging RCDs for ischemic stroke therapy, clinical translation still faces multiple challenges: (1) Temporal specificity: The dominant death modalities differ across the acute (0–72 h), subacute (3 days–6 weeks), and chronic (>6 weeks) phases, requiring the development of phase-adaptive therapies (Candelario-Jalil et al., 2022). (2) Cell type selectivity: Neurons, microglia, astrocytes, and endothelial cells exhibit varying sensitivities to the same death pathway, necessitating cell-specific targeting (Wang C. et al., 2024). (3) Blood-brain barrier penetration: The BBB permeability of most small-molecule inhibitors is limited. MCC950, for example, has an estimated CNS-to-plasma ratio of <0.1 in naive rodents, and its apparent brain exposure further varies with post-ischemic BBB disruption, which peaks at 24–72 h and partially recovers by day 7 (Kadry et al., 2020). Strategies to overcome this include: engineered PLGA nanoparticles with surface functionalization for receptor-mediated transcytosis, exosome-based carriers with inherent BBB-crossing capability, and intranasal delivery, which bypasses the BBB via the olfactory/trigeminal route and has shown promise for delivering NLRP3 inhibitors and caspase inhibitors in rodent stroke models. Each strategy has distinct pharmacokinetic profiles that must be validated in non-human primate models before clinical application. (4) Biomarker development: Serum copper levels, cysteine/cystine ratios, and IL-1β/IL-18 levels may serve as clinical biomarkers of RCD activation, but real-time intracerebral monitoring technology remains immature (Jin et al., 2021). (5) Personalized medicine: Precision typing based on genomics (e.g., PRDX1 polymorphisms) and metabolomics can guide individualized RCD-targeted therapy (Liu et al., 2024).

## 7 Conclusion

Cuproptosis, disulfidptosis, and PANoptosis, as three emerging regulated cell death modalities, play important roles in the pathological progression of ischemic stroke. Cuproptosis directly injures neurons through the copper ion–lipoylated protein–mitochondrial dysfunction axis; disulfidptosis kills cells through the SLC7A11–NADPH–actin cytoskeletal collapse mechanism in the glucose-deprived microenvironment characteristic of ischemia; and PANoptosis amplifies neuroinflammation and secondary injury through the PANoptosome integration of pyroptosis, apoptosis, and necroptosis. These three death modalities do not exist in isolation but form complex interaction networks through oxidative stress, mitochondrial metabolism, and inflammatory signaling.

Future research should focus on: (1) utilizing single-cell multi-omics and spatial transcriptomics to resolve the RCD spectrum characteristics of different NVU cells at different phases of stroke; (2) developing targeted drugs with BBB penetration and cell selectivity; (3) establishing patient stratification systems based on RCD biomarkers to achieve precision intervention; and (4) exploring synergistic therapeutic strategies that simultaneously target multiple RCDs. With deepening understanding of emerging RCD mechanisms and advances in translational medicine technologies, targeting cuproptosis, disulfidptosis, and PANoptosis is expected to open new avenues for neuroprotective therapy in ischemic stroke. Looking further ahead, we propose that the mechanistic convergence of cuproptosis, disulfidptosis, and PANoptosis on shared metabolic and oxidative hubs—particularly the mitochondrial ROS–NADPH–GSH axis—argues for developing a unified ‘metabolic cell death’ conceptual framework that treats these modalities not as parallel but as dynamically interconverting states governed by real-time cellular energy status. Testing this integrative model should be an explicit priority for future mechanistic work. Furthermore, the advent of spatially resolved single-cell transcriptomics and live-cell metabolic imaging now makes it feasible, for the first time, to map the precise co-occurrence and sequential activation of these three death programs within individual neurovascular units across the ischemic territory in real time—a methodological Frontier that we anticipate will fundamentally redefine our understanding of secondary injury progression in stroke and catalyze the next-generation of RCD-targeted neuroprotective therapies.

## Glossary

IS: Ischemic stroke
RCD: Regulated cell death
NVU: Neurovascular unit
OGD: Oxygen-glucose deprivation
CIRI: Cerebral ischemia-reperfusion injury
BBB: Blood-brain barrier
CTR1: Copper transporter 1 (SLC31A1)
FDX1: Ferredoxin 1
LIAS: Lipoic acid synthetase
DLAT: Pyruvate dehydrogenase complex E2 subunit
SLC7A11: Solute carrier family 7, member 11 (System Xc^−^ catalytic subunit)
GPX4: Glutathione peroxidase 4
GSH: Glutathione
NADPH: Nicotinamide adenine dinucleotide phosphate (reduced form)
PPP: Pentose phosphate pathway
PRDX1: Peroxiredoxin 1
ZBP1: Z-DNA binding protein 1
NLRP3: NLR family pyrin domain-containing protein 3
AIM2: Absent in melanoma 2
GSDMD: Gasdermin D
MLKL: Mixed lineage kinase domain-like protein
RIPK1/3: Receptor-interacting protein kinase 1/3
FADD: Fas-associated death domain protein
ASC: Apoptosis-associated speck-like protein containing a CARD
mtDNA: Mitochondrial DNA
mPTP: Mitochondrial permeability transition pore
TTM/ATTM: Ammonium tetrathiomolybdate
SAS: Sulfasalazine
TCEP: Tris(2-carboxyethyl)phosphine
DTT: Dithiothreitol
NAC: N-acetylcysteine
Nec-1s: Necrostatin-1s
2-DG: 2-deoxy-D-glucose
PLGA: Poly(lactic-co-glycolic acid)
